# Efficacy of budesonide/formoterol maintenance and reliever therapy compared with higher-dose budesonide as step-up from low-dose inhaled corticosteroid treatment

**DOI:** 10.1186/s12890-017-0401-y

**Published:** 2017-04-20

**Authors:** Christine R. Jenkins, Göran Eriksson, Eric D. Bateman, Helen K. Reddel, Malcolm R. Sears, Magnus Lindberg, Paul M. O’Byrne

**Affiliations:** 1 Department of Thoracic Medicine, Concord Hospital and The George Institute for Global Health, PO Box M201, Missenden Rd, Sydney, NSW 2050 Australia; 2 Department of Respiratory Medicine and Allergology, University Hospital, Lund, Sweden; 3 Division of Pulmonology, Department of Medicine, University of Cape Town, Cape Town, South Africa; 4 Clinical Management Group, Woolcock Institute of Medical Research, University of Sydney, Sydney, Australia; 5 Michael G DeGroote School of Medicine, Faculty of Health Sciences, McMaster University, Hamilton, Canada; 6 Biometrics and Information Sciences (B&I), AstraZeneca R&D, Mölndal, Sweden

**Keywords:** Asthma, Bronchodilators, Corticosteroids, Respiratory therapy, Therapy

## Abstract

**Background:**

Asthma management may involve a step up in treatment when symptoms are not well controlled. We examined whether budesonide/formoterol maintenance and reliever therapy (MRT) is as effective as higher, fixed-dose budesonide plus as-needed terbutaline in patients requiring step-up from Step 2 treatment (low-dose inhaled corticosteroids), stratified by baseline reliever use.

**Methods:**

A *post-hoc* analysis utilized data from three clinical trials of 6–12 months’ duration. Patients aged ≥12 years with symptomatic asthma uncontrolled despite Step 2 treatment were included. Severe exacerbation rate, lung function and reliever use were analysed, stratified by baseline reliever use (<1, 1–2 and >2 occasions/day).

**Results:**

Overall, 1239 patients were included. Reductions in severe exacerbation rate with budesonide/formoterol MRT versus fixed-dose budesonide were similar across baseline reliever use levels, and were statistically significant in patients using 1–2 (42%, *p* = 0.01) and >2 (39%, *p* = 0.02) reliever occasions/day, but not <1 reliever occasion/day (35%, *p* = 0.11). Both treatments significantly increased mean FEV_1_ from baseline; improvements were significantly greater for budesonide/formoterol MRT in all reliever use groups. Reductions in reliever use from baseline were significantly greater with budesonide/formoterol MRT versus fixed-dose budesonide in patients using 1–2 and >2 reliever occasions/day (−0.33 and −0.74 occasions/day, respectively).

**Conclusions:**

Treatment benefit with budesonide/formoterol MRT versus higher, fixed-dose budesonide plus short-acting β_2_-agonist was found in Step 2 patients with relatively low reliever use, supporting the proposal that budesonide/formoterol MRT may be useful when asthma is uncontrolled with low-dose inhaled corticosteroid.

**Electronic supplementary material:**

The online version of this article (doi:10.1186/s12890-017-0401-y) contains supplementary material, which is available to authorized users.

## Background

Mild asthma comprises approximately 70% of asthma in the community [1], and accounts for a significant economic burden [2]. Despite this, the principal focus of most research has been in patients with more severe asthma. The long-term goals of asthma management are to achieve good control of symptoms and to minimise future risk to the patient, including exacerbations [3]. Whilst poor symptom control is a well-known predictor of risk of exacerbations [4], even patients with well controlled asthma symptoms may continue to experience exacerbations, and require interventions to reduce this risk.

The Global Initiative for Asthma (GINA) report recommends control-based asthma management, with pharmacological and non-pharmacological treatments adjusted in a continuous cycle based on assessment of symptom control and risk factors; a step up in treatment is recommended if asthma symptoms are not well controlled or if exacerbations continue despite good adherence and correct technique with the patient’s existing inhaled treatment [3]. The recommended treatment at Step 2 is low-dose inhaled corticosteroid (ICS) as maintenance treatment plus as-needed short-acting β_2_-agonist (SABA) as reliever medication [3]. It was established in the 1990s that, for those needing a step up in treatment, maintenance therapy with a low-dose ICS/long-acting β_2_-agonist (ICS/LABA) plus as-needed SABA as reliever was more effective than a two- or four-fold higher dose of ICS [5, 6]; hence, the preferred Step 3 options are either low-dose ICS/formoterol, such as budesonide/formoterol (BUD/FORM), as both maintenance and reliever therapy (MRT), or conventional maintenance treatment with low-dose ICS/LABA plus as-needed SABA as reliever. An alternative Step 3 option is a two- or even four-fold higher dose of ICS plus as-needed SABA as reliever [3]. The latter is equally recommended with maintenance low-dose ICS/LABA in the US guidelines (Expert Panel Report 3) [7], and is the recommended Step 3 treatment in the International Union Against Tuberculosis and Lung Disease guidelines [8].

We have previously shown in a *post-hoc* analysis of five studies that, in patients with sub-optimal asthma control despite GINA Step 2, 3 or 4 treatment, BUD/FORM MRT is more effective in improving symptom control and reducing exacerbations than higher-dose ICS [9]. However, most patients in these studies had poor symptom control at entry, with mean reliever use of 1.7 to 2.4 occasions/day, which is well above the criterion of SABA use of ≥3 occasions/week at which a step-up would normally be considered.

Thus, it is relevant to question whether BUD/FORM MRT is effective in patients with milder, less poorly controlled disease at study entry, i.e. those with less frequent reliever use while taking low-dose ICS (≤400 μg/day BUD equivalent). This *post-hoc* analysis evaluated the efficacy of BUD/FORM MRT in improving exacerbation rate, lung function and reliever use compared with reference treatment of a higher, fixed dose of BUD plus as-needed SABA in such patients, stratified by baseline reliever use.

## Methods

### Study design

This retrospective, *post-hoc* analysis included data from double-blind, randomized, parallel-group studies of 6 [10] and 12 [11, 12] months’ duration. The detailed methodologies have been published elsewhere [10–12]. Briefly, the clinical studies investigated the efficacy of BUD/FORM 160–320/9 μg/day MRT (Symbicort SMART™; AstraZeneca, Lund, Sweden) compared with fixed-dose BUD 320–640 μg/day as maintenance therapy and the SABA, terbutaline 0.4 mg, as needed. A fixed dose of BUD/FORM 160/9 μg maintenance therapy plus terbutaline 0.4 mg as-needed treatment arm was investigated as a comparator only in one [12] of three studies and was not included in the present analysis for this reason. All study drugs were administered using Turbuhaler® (AstraZeneca, Lund, Sweden) dry powder inhaler as delivered doses. Patients were permitted to take a maximum of 10 as-needed occasions/day of BUD/FORM or terbutaline before contacting the investigator for reassessment. The studies were performed in accordance with the Declaration of Helsinki and Good Clinical Practice guidelines. Approval from regulatory agencies and ethics committees was obtained at all centres. All patients gave written informed consent.

### Patients

Patients aged 12–80 [10, 11] and 4–80 [12] years and with a diagnosis of asthma were enrolled in the clinical studies. Inclusion criteria comprised: a forced expiratory volume in 1 s (FEV_1_) of 60–100% predicted in two studies [10, 12], and FEV_1_ 50–90% predicted in the third study [11]; baseline bronchodilator reversibility of ≥12%; and, in two studies [11, 12], at least one exacerbation in the 12 months prior to enrolment. Patients were required to have a minimum of 7 [10] and 12 [11, 12] as-needed occasions of terbutaline during the last 10 days of run-in for enrolment, but no more than 10 occasions on any day. All patients received ICS (200–1600 μg/day) for ≥3 months and at a constant dose for 30 days prior to study entry.

In the present *post-hoc* analysis, patients with asthma aged ≥12 years who were receiving Step 2 treatment (low-dose ICS, ≤400 μg/day BUD equivalent, and no long-acting β_2_ agonist therapy) were included. Outcomes were analysed across a range of baseline reliever use levels: <1, 1–2 and >2 occasions/day. These cut-points were chosen for clinical simplicity rather than based on statistical distribution.

### Efficacy evaluations

A severe exacerbation was defined as hospitalization/emergency department treatment due to asthma worsening or the need for oral steroid treatment of asthma (as judged by the investigator). FEV_1_ measurements were assessed by spirometry at enrolment and all clinic visits, and in accordance with the European Respiratory Society recommendations [13]. Use of reliever medication (terbutaline or BUD/FORM according to randomised allocation) was recorded by patients using diary cards.

### Statistical analysis

Efficacy analysis was carried out for each study variable to determine whether BUD/FORM MRT was more efficacious than fixed-dose BUD by baseline reliever use (<1, 1–2 and >2 occasions/day). Severe exacerbation rates were analysed using Poisson regression with treatment and study as factors, and presented as p values and 95% confidence intervals (CI). On-treatment FEV_1_ [14] and reliever use were analysed as treatment average change from baseline using analysis of covariance (ANCOVA) with treatment, study and baseline as fixed factors; least-square mean [LSM] treatment differences and 95% CI were calculated. In addition, the proportion of patients on both treatments with baseline reliever use ≥1 and >2 occasions/day who achieved a reduction in mean reliever use to thresholds of <0.5 and <1 occasion/day was analysed using Fisher’s exact test; patients with baseline reliever use ≥1 occasions/day comprise those from both the 1–2 and >2 occasions/day baseline reliever use subgroups. P values <0.05 were considered statistically significant. Statistical analysis was performed using standard statistical software (SAS v9.2, SAS Institute Inc., NC, USA).

## Results

### Patients

In total, 1239 patients (BUD/FORM MRT = 626; fixed-dose BUD = 613) were included in this *post-hoc* analysis. Baseline data for these patients are presented in Table 1. Baseline characteristics between randomization groups were comparable, including lung function, reliever use and ICS dose, but as expected baseline lung function was lower in the groups with highest baseline reliever use (Table 1).

**Table 1:** Patients’ baseline and demographic data, stratified by baseline reliever use (<1, 1–2 and >2 occasions/day)

	BUD/FORM MRT (All patients) (*n* = 626)	Fixed-dose BUD (All patients) (*n* = 613)	Baseline reliever use subgroup					
			<1 occasion/day		1–2 occasions/day		>2 occasions/day	
			BUD/FORM MRT (*n* = 168)	Fixed-dose BUD (*n* = 155)	BUD/FORM MRT (*n* = 257)	Fixed-dose BUD (*n* = 253)	BUD/FORM MRT (*n* = 201)	Fixed-dose BUD (*n* = 205)
Male, n (%)	254 (40.6)	227 (37.0)	71 (42.3)	58 (37.4)	104 (40.5)	100 (39.5)	79 (39.3)	69 (33.7)
Age, year	37.0 (15.9)	38.3 (16.5)	35.6 (15.7)	37.5 (16.0)	36.0 (16.2)	37.7 (17.0)	39.4 (15.5)	39.6 (16.1)
Duration of asthma, years^a^	10	9	7	8	10	10	11	10
Dose of inhaled corticosteroid, μg/day BUD eqv	357.8 (70.2)	356.1 (70.8)	339.9 (84.6)	325.4 (88.1)	359.9 (71.0)	367.2 (63.2)	370.1 (51.0)	365.6 (57.4)
Pre-BD FEV_1_ % predicted	73.6 (10.9)	73.0 (10.7)	74.0 (11.2)	74.0 (11.1)	74.8 (10.6)	73.4 (10.6)	71.7 (10.6)	71.7 (10.5)
Post-BD FEV_1_ % predicted	88.5 (12.6)	88.0 (12.9)	89.4 (12.9)	89.0 (13.6)	89.5 (11.7)	88.5 (12.4)	86.5 (13.2)	86.8 (12.8)
Baseline pre-BD FEV_1_, L	2.41 (0.7)	2.35 (0.7)	2.51 (0.7)	2.47 (0.7)	2.46 (0.7)	2.41 (0.8)	2.26 (0.7)	2.19 (0.7)
Baseline FEV_1_ reversibility, %	14.9 (7.5)	15.0 (7.8)	15.4 (7.0)	15.0 (8.0)	14.7 (7.6)	15.0 (7.9)	14.9 (7.9)	15.1 (7.6)
Current smoker/ex-smoker, n (%)	117 (18.7)	114 (18.6)	26 (15.5)	24 (15.5)	52 (20.2)	47 (18.6)	39 (19.4)	43 (21.0)

Data are given as mean (SD), unless otherwise stated

Patients treated with BUD/FORM MRT comprised 254 patients from the STEAM study, [10] 171 patients from the STEP study, [11] and 201 patients from the STAY study [12]

Patients treated with fixed-dose BUD comprised 255 patients from the STEAM study, [10] 161 patients from the STEP study, [11] and 197 patients from the STAY study [12]

^a^Median

*BD* bronchodilator, *BUD* budesonide, *eqv* equivalent, *FEV*_*1*_ forced expiratory volume in 1 s, *FORM* formoterol, *MRT* maintenance and reliever therapy, *SD* standard deviation

### Severe exacerbations

The percentage of patients with a severe exacerbation generally increased with increasing baseline reliever use for both treatments (Table 2). In all baseline reliever use groups, the percentage of patients with a severe exacerbation was lower with BUD/FORM MRT than fixed-dose BUD (Table 2).

**Table 2:** Clinical outcomes for patients treated with BUD/FORM MRT or fixed-dose BUD, stratified by baseline reliever use (<1, 1–2 and >2 occasions/day)

	BUD/FORM MRT (All patients) (*n* = 626)	Fixed-dose BUD (All patients) (*n* = 613)	Baseline reliever use subgroup					
			<1 occasion/day		1–2 occasions/day		>2 occasions/day	
			BUD/FORM MRT (*n* = 168)	Fixed-dose BUD (*n* = 155)	BUD/FORM MRT (*n* = 257)	Fixed-dose BUD (*n* = 253)	BUD/FORM MRT (*n* = 201)	Fixed-dose BUD (*n* = 205)
Severe exacerbations								
Patients with severe exacerbation, n (%)	38 (6.1)	72 (11.7)	8 (4.8)	11 (7.1)	11 (4.3)	24 (9.5)	19 (9.5)	37 (18.0)
Rate, exacerbations per year	0.14	0.23	0.09	0.14	0.09	0.16	0.22	0.37
FEV_1_								
FEV_1_, L	2.62 (0.8)	2.46 (0.7)	2.69 (0.7)	2.55 (0.7)	2.66 (0.8)	2.50 (0.8)	2.51 (0.8)	2.34 (0.7)
Change from baseline in FEV_1_, L	0.21 (0.3)	0.11 (0.3)	0.18 (0.3)	0.08 (0.3)	0.20 (0.3)	0.10 (0.3)	0.25 (0.4)	0.16 (0.4)
Reliever use								
Treatment period reliever use, occasions/day	0.84 (1.2)	1.27 (1.5)	0.43 (0.7)	0.50 (0.8)	0.60 (0.7)	0.93 (1.0)	1.50 (1.7)	2.28 (1.7)
Change from baseline in reliever use, occasions/day	−1.00 (1.3)	−0.65 (1.3)	−0.15 (0.7)	−0.15 (0.8)	−0.89 (0.8)	−0.56 (1.0)	−1.84 (1.6)	−1.14 (1.7)

Data are given as mean (SD), unless otherwise stated

*BUD* budesonide, *FORM* formoterol, *MRT* maintenance and reliever therapy

**Fig. 1:**
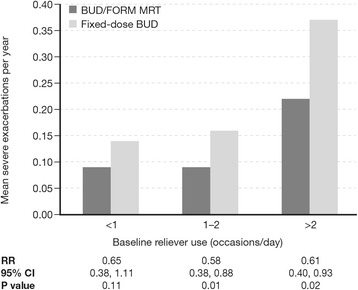
Severe exacerbation rates by treatment in patients with baseline reliever use (<1, 1–2 and >2 occasions/day). Rate ratios (95% CI) and statistical comparisons are for BUD/FORM MRT compared with fixed-dose BUD. *BUD* budesonide, *CI* confidence intervals, *FORM* formoterol, *MRT* maintenance and reliever therapy, *RR* rate ratio

Severe exacerbation rates for patients treated with BUD/FORM MRT and fixed-dose BUD stratified by baseline reliever use levels are shown in Table 2. In the group using reliever on <1 occasion/day at baseline, there was a 35% reduction in severe exacerbations for BUD/FORM MRT versus fixed-dose BUD (mean 0.09 vs 0.14 severe exacerbations per year, respectively; Table 2), but this effect did not reach statistical significance (rate ratio = 0.65 [95% CI: 0.38, 1.11]; *p* = 0.11; Fig. 1). In the baseline reliever use 1–2 occasions/day group, exacerbation rates for patients treated with BUD/FORM MRT and fixed-dose BUD were mean 0.09 and 0.16 exacerbations per year, respectively (Table 2), corresponding with a 42% reduction in severe exacerbations (rate ratio = 0.58 [95% CI: 0.38, 0.88]; *p* = 0.01; Fig. 1) for BUD/FORM MRT compared with fixed-dose BUD. Among patients with reliever use >2 occasions/day at baseline, patients treated with BUD/FORM MRT had fewer severe exacerbations than those treated with fixed-dose BUD (mean 0.22 vs 0.37 exacerbations per year, Table 2), corresponding with a 39% lower reduction in severe exacerbations (rate ratio = 0.61 [95% CI: 0.40, 0.93]; *p* = 0.02; Fig. 1).

### FEV_1_

**Fig. 2:**
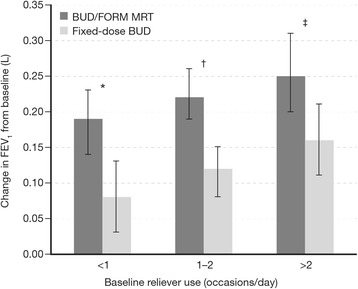
Least square mean improvements in mean FEV_1_ from baseline in patients treated with BUD/FORM MRT or fixed-dose BUD, stratified by baseline reliever use (<1, 1–2 and >2 occasions/day). The error bars represent 95% CI. Statistical comparisons are for BUD/FORM MRT compared with fixed-dose BUD. *BUD* budesonide, *CI* confidence intervals, *FORM* formoterol, *FEV*_*1*_ forced expiratory volume in 1 s, *MRT* maintenance and reliever therapy. * +0.11 L (95% CI: 0.04, 0.17), *p* = 0.001; † +0.11 L (95% CI: 0.06, 0.16), *p* < 0.0001; ‡ +0.10 L (95% CI: 0.02, 0.17), *p* = 0.01

Both BUD/FORM MRT and fixed-dose BUD significantly improved mean FEV_1_ from baseline in all three reliever use groups; for both treatments, improvements from baseline were greatest in patients with baseline reliever use >2 occasions/day (Table 2). For all reliever use groups, improvements from baseline in mean FEV_1_ were significantly greater for BUD/FORM MRT compared with fixed-dose BUD (treatment differences: <1 occasion/day = +0.11 L [95% CI: 0.04, 0.17], *p* = 0.001; 1–2 occasions/day = +0.11 L [95% CI: 0.06, 0.16], *p* < 0.0001; >2 occasions/day = +0.10 L [95% CI: 0.02, 0.17], *p* = 0.01; Fig. 2).

### Reliever use

**Fig. 3:**
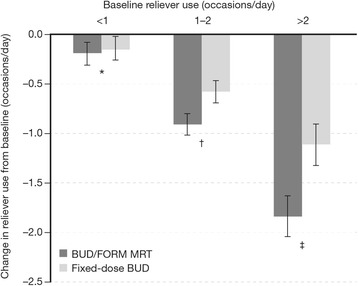
Least square mean improvements in on-treatment reliever use from baseline in patients treated with BUD/FORM MRT or fixed-dose BUD, stratified by baseline reliever use (<1, 1–2 and >2 occasions/day). The error bars represent 95% CI. Statistical comparisons are for BUD/FORM MRT compared with fixed-dose BUD. *BUD* budesonide, *CI* confidence intervals, *FORM* formoterol, *MRT* maintenance and reliever therapy. * –0.03 occasions/day (95% CI: −0.20, 0.13), *p* = 0.68; † −0.33 occasions/day (95% CI: −0.48, −0.18), *p* < 0.0001; ‡ −0.74 occasions/day (95% CI: −1.03, −0.45), *p* < 0.0001

Both BUD/FORM MRT and fixed-dose BUD reduced reliever use from baseline in all three baseline reliever use groups (Table 2); reductions were greater with increasing baseline reliever use and were most prominent in patients using >2 occasions/day. In patients using <1 reliever occasion/day at baseline, reductions in reliever use for BUD/FORM MRT compared with fixed-dose BUD were not statistically significant; treatment difference = -0.03 occasions/day, *p* = 0.68 (Table 2, Fig. 3). Among patients using 1–2 and >2 reliever occasions/day at baseline, reductions in mean reliever use were significantly greater for BUD/FORM MRT than fixed-dose BUD; 1–2 occasions/day: treatment difference = -0.33 occasions/day, *p* < 0.0001; >2 occasions/day: treatment difference = -0.74 occasions/day, *p* < 0.0001 (Table 2, Fig. 3).

For patients with baseline reliever use ≥1 and >2 occasions/day, the proportion of patients who achieved a reduction in mean reliever use to thresholds of <0.5 and <1 occasion/day was significantly greater with BUD/FORM MRT than fixed-dose BUD (all *p* < 0.0001, Additional file 1: Table S1).

## Discussion

The efficacy of BUD/FORM MRT for reducing exacerbations in at-risk patients with uncontrolled asthma across a range of baseline treatment steps is well established [9]. The present *post-hoc* analysis, based on patients using Step 2 treatment (low-dose ICS, ≤400 μg/day BUD equivalent) at entry into three double-blind, randomized, parallel-group studies [10–12], and stratifying by baseline reliever use (<1, 1–2 and >2 occasions/day), suggests that the benefit from MRT is seen even in patients with milder asthma and impaired asthma control, most of whom had a history of at least one exacerbation in the previous year. The magnitude of difference in severe exacerbations for MRT compared with higher-dose ICS was similar regardless of baseline reliever use, reducing the proportion of patients having a severe exacerbation by 35–42%; however, statistical significance was not reached in the lowest stratum. Furthermore, this reduction in exacerbation risk with BUD/FORM MRT was achieved with a maintenance dose of ICS approximately two-[10, 11] to four-[12] fold lower than with fixed-dose BUD. This finding supports the value of MRT treatment in this milder category of asthma in terms of reducing exacerbation risk. Importantly, exacerbation reduction may still be important in this group of patients with low exacerbation rates since a significant proportion of asthma deaths occur in patients with so-called mild asthma [15–17].

This study also considers a problem common in clinical practice, that is, the situation in which patients use higher than recommended levels of reliever use before they or their physicians step up treatment [18]. The recommended approach in patients with mild asthma who remain uncontrolled and require reliever medication ≥3 occasions/week [3] despite maintenance treatment with low-dose ICS (<400 μg/day BUD equivalent) and as-needed SABA as reliever, is to check adherence and inhaler technique, and then to step up treatment to low-dose ICS/formoterol (budesonide or beclomethasone) MRT *or* low-dose maintenance ICS/LABA plus as-needed SABA [3]. A third option is medium-dose ICS plus as-needed SABA as reliever. Frequent reliever use is a marker for sub-optimal asthma control and is associated with an increased risk of an exacerbation [4]. The latter has been confirmed in many studies, and a recent comparison of BUD/FORM MRT with conventional maintenance BUD/FORM and as-needed SABA showed that baseline reliever use was a significant predictor of severe exacerbations within the following 12 months [19].

Improvements in measures of daily asthma control (lung function, reliever use) were also seen for BUD/FORM MRT compared with fixed-dose BUD. Given that the comparator was ICS alone, it is not surprising that improvements in on-treatment FEV_1_ [14] from baseline were significantly greater for BUD/FORM MRT than fixed-dose BUD (treatment differences 0.10–0.11 L). However, although statistically significant, improvements in FEV_1_ of 0.10–0.11 L are relatively small despite these patients having mean 15% FEV_1_ reversibility and low prebronchodilator FEV_1_ at baseline. That the magnitude of reduction in reliever use with BUD/FORM MRT was smallest in patients with lower baseline reliever use suggests that these patients may have milder underlying asthma or may disregard some symptoms, or that reliever use at such low levels may be a relatively insensitive marker of treatment effect. Despite this, our exacerbation data suggest that there is still potential for clinical benefit by reducing their risk of adverse outcomes. Further to the mean changes in reliever use, BUD/FORM MRT significantly increased the proportion of patients with baseline usage ≥1 and >2 occasions/day whose reliever use fell below 0.5 occasions/day (3.5 occasions/week), usage above which a step-up in treatment would normally be considered.

One mechanism by which BUD/FORM MRT may reduce exacerbations even in patients with infrequent reliever use is by improving adherence with ICS-containing medication. Poor adherence is associated with significant asthma-related morbidity but, despite this, patients with milder asthma may not take daily maintenance treatment on symptom-free days [20, 21], which supports the rationale for the use of MRT in patients with reliever use of <1 occasion/day. The use of a single combination inhaler for ICS/LABA MRT simplifies asthma management and ensures that reliever treatment provides both immediate symptom relief and a rapid anti-inflammatory effect, preventing symptoms from developing into an exacerbation. There is also an established scientific rationale for giving an ICS and a LABA together as they have complementary actions on the pathophysiology of asthma and may act synergistically at a molecular level [22, 23].

We acknowledge that the *post-hoc* design of this analysis is a limitation and future prospective, well-designed trials are required to confirm these findings in patients with milder asthma and lower baseline reliever use. The use of ICS/LABA as-needed in mild asthma is currently being evaluated in the ongoing SYGMA studies [24]. In the present analysis, the selection only of patients on Step 2 treatment at entry, and sub-division by baseline as-needed reliever use, reduced the power of the analyses, with only around 150–250 patients qualifying in each sub-group. Additionally, all patients satisfied the regulatory requirement for significant bronchodilator reversibility at entry, and the studies had minimum reliever use requirements of 7 [10] and 12 [11, 12] occasions in the last 10 days of run-in, so the results cannot and should not be extrapolated to patients with well controlled asthma (e.g., with reliever use <3 occasions/week). Finally, patients in two of the studies were required to have had at least one exacerbation in the previous 12 months and may thus represent a group of patients at higher risk than others at this step of treatment.

## Conclusions

In conclusion, BUD/FORM MRT and a higher, fixed dose of BUD plus SABA improved lung function, and reduced reliever use and exacerbation rate, in patients whose asthma was uncontrolled on Step 2 treatment at study entry. Treatment benefit for exacerbations with BUD/FORM MRT compared with a higher, fixed-dose BUD plus SABA was found in these patients across all levels of baseline reliever use, and was statistically significant in those with a baseline reliever use of 1–2 and >2 occasions/day. These results support the beneficial effects of BUD/FORM MRT in patients whose asthma is not well-controlled with low-dose ICS, even when their background use of reliever is relatively low.

## Additional file

Additional file 1: Table S1. Proportion of patients with baseline reliever use ≥1 and >2 occasions/day who achieved mean reliever use thresholds of <1 and <0.5 occasion/day following treatment with BUD/FORM MRT or fixed-dose BUD. (DOCX 14 kb)

## Abbreviations

BD: Bronchodilator
BUD/FORM: Budesonide/formoterol
CI: Confidence interval
FEV_1_: Forced expiratory volume in 1 second
GINA: Global Initiative for Asthma
ICS: Inhaled corticosteroids
LABA: Long-acting β_2_-agonist
MRT: Maintenance reliever therapy
RR: Rate ratio
SABA: Short-acting β_2_-agonist
SD: Standard deviation
