# Supplementary material for: Efficacy of budesonide/formoterol maintenance and reliever therapy compared with higher-dose budesonide as step-up from low-dose inhaled corticosteroid treatment
Source: BMC Pulm Med. 2017 Apr 20;17:65. doi: 10.1186/s12890-017-0401-y (PMC5397768; doi:10.1186/s12890-017-0401-y)
Supplement: Additional file 1: Table S1. — Proportion of patients with baseline reliever use ≥1 and >2 occasions/day who achieved mean reliever use thresholds of <1 and <0.5 occasion/day following treatment with BUD/FORM MRT or fixed-dose BUD. (DOCX 14 kb) [file 12890_2017_401_MOESM1_ESM.docx]

**Supplementary Data**

***Additional file 1 Table S1***

Proportion of patients with baseline reliever use ≥1 and >2 occasions/day who achieved mean reliever use thresholds of <1 and <0.5 occasion/day following treatment with BUD/FORM MRT or fixed-dose BUD.

|  | Baseline reliever use | | | |
| --- | --- | --- | --- | --- |
|  | **≥1 occasions/day*** | | **>2 occasions/day** | |
|  | BUD/FORM MRT  (n = 458) | Fixed-dose BUD  (n = 458) | BUD/FORM MRT  (n = 201) | Fixed-dose BUD  (n = 205) |
| Patients who achieved mean reliever use, n (%) | | | | |
| <1 occasion/day | 303 (66.2)† | 215 (46.9) | 103 (51.2)† | 54 (26.3) |
| <0.5 occasion/day | 227 (49.6)† | 149 (32.5) | 72 (35.8)† | 29 (14.2) |

* Patients with baseline reliever use ≥1 occasions/day comprise those from both the 1–2 and >2 occasions/day baseline reliever use subgroups.

† p<0.0001 for BUD/FORM MRT versus fixed-dose BUD.

BUD, budesonide; FORM, formoterol; MRT, maintenance and reliever therapy.
